# Transforming growth factor-induced gene TGFBI is correlated with the prognosis and immune infiltrations of breast cancer

**DOI:** 10.1186/s12957-024-03301-z

**Published:** 2024-01-20

**Authors:** Haiwei Wang, Xinrui Wang, Liangpu Xu

**Affiliations:** Medical Genetic Diagnosis and Therapy Center, Fujian Key Laboratory for Prenatal Diagnosis and Birth Defect, Fujian Maternity and Child Health Hospital, Fujian Medical University, Fuzhou, Fujian China

**Keywords:** Breast cancer, TGFβ, TGFBI, Prognosis, Immune infiltrations

## Abstract

**Background:**

Transforming growth factor β (TGFβ) is a critical regulator of lung metastasis of breast cancer and is correlated with the prognosis of breast cancer. However, not all TGFβ stimulated genes were functional and prognostic in breast cancer lung metastatic progress. In this study, we tried to determine the prognosis of TGFβ stimulated genes in breast cancer.

**Methods:**

TGFβ stimulated genes in MDA-MB-231 cells and lung metastasis-associated genes in LM2-4175 cells were identified through gene expression microarray. The prognosis of the induced gene (TGFBI) in breast cancer was determined through bioinformatics analysis and validated using tissue microarray. The immune infiltrations of breast cancer were determined through “ESTIMATE” and “TIMER”.

**Results:**

TGFBI was up-regulated by TGFβ treatment and over-expressed in LM2-4175 cells. Through bioinformatics analysis, we found that higher expression of TGFBI was associated with shorted lung metastasis-free survival, relapse-free survival, disease-free survival, and overall survival of breast cancer. Moreover, the prognosis of TGFBI was validated in 139 Chinese breast cancer patients. Chinese breast cancer patients with higher TGFBI expression had lower overall survival. Correspondingly, breast cancer patients with higher TGFBI methylation had higher overall survival. TGFBI was correlated with the score of the TGFβ signaling pathway and multiple immune-related signaling pathways in breast cancer. The stromal score, immune score, and the infiltrations of immune cells were also correlated with TGFBI expression in breast cancer.

**Conclusions:**

TGFβ-induced gene TGFBI was correlated with the prognosis and immune infiltrations of breast cancer.

**Supplementary Information:**

The online version contains supplementary material available at 10.1186/s12957-024-03301-z.

## Background

Breast cancer is the most common malignancy in women and is a heterogeneous disease [1]. According to the expression levels of estrogen receptor (ER), progesterone receptor (PR), human epidermal growth factor receptor 2 (HER2), and Ki67, breast cancer is classified into Luminal A, Luminal B, HER2 over-expression and Basal-like (Triple negative) sub-types [2]. Triple-negative breast cancer cells are highly metastatic and induce high mortality [3]. Lung is the most common metastatic organ of triple-negative breast cancer cells [4]. The pulmonary capillaries could provide sufficient oxygen for the rapid proliferation of tumor cells. Also, the lung tissues contain a large number of alveolar macrophages that secrete various cytokines, including transforming growth factor β (TGFβ) to increase vascular permeability, promote tumor angiogenesis, and then promote tumor growth and metastasis [5].

TGFβ is a multifunctional cytokine which regulates tissue and embryo development, controls cell growth and differentiation, induces immune and inflammatory response, and participates in angiogenesis, tumor growth, metastasis, and other important biological processes [6]. The expressions of TGFβ stimulated genes are regulated by downstream SMAD transcription factors [7]. In the early stage of tumorigenesis, TGFβ inhibits the proliferation of tumor cells and induces apoptosis [8]. However, at the metastatic stage, tumor cells are no longer responsive to TGFβ induced growth inhibition. On the contrary, upon the stimulation of TGFβ, the tumor cells become more aggressive. In addition, TGFβ can be induced under hypoxia and inflammation conditions and has protective effects on tumor cells [9]. TGFβ could also drive epithelial-mesenchymal transformation (EMT) of tumor cells and enhance their metastatic ability [10].

TGFβ also plays a key roles in the metastatic progresses of breast cancer cells. In ER-negative breast cancer patients, TGFβ signal pathway is related to lung metastasis, but not to bone metastasis of breast cancer [7]. Blocking the TGFβ signal pathway inhibits the lung metastasis of breast cancer, but does not affect the growth of breast cancer cells [7]. Angiopoietin-like 4 (ANGPTL4) is an important TGFβ downstream factor and is associated with the prognosis of breast cancer [11, 12]. Functionally, ANGPTL4 destroys the connections between vascular endothelial cells, enhancing the entering of breast cancer cells into the lung parenchyma [7]. Blocking the expressions of ANGPTL4 inhibits lung metastasis of breast cancer cells. TGFβ stimulated genes matrix metallopeptidase 2 (MMP2) [13], epiregulin (EREG) [14], and vascular endothelial growth factor A (VEGFA) [15] were also critical regulators of lung metastasis of breast cancer and correlated with the prognosis of breast cancer.

However, not all TGFβ stimulated genes are functional and prognostic in the breast cancer lung metastatic progress. Revealing the detailed functions and prognosis of TGFβ stimulated genes will provide more understanding of the TGFβ signal in breast cancer. In this study, we identified the TGFβ stimulated genes in MDA-MB-231 cells and lung metastasis-associated genes in LM2-4175 cells [16]. We showed that the TGFβ-induced gene (TGFBI) was upregulated by TGFβ treatment and over-expressed in LM2-4175 cells. Moreover, TGFBI was correlated with the prognosis and immune infiltrations of breast cancer.

## Methods

### Cell culture

LM2-4175 is a sub-clone of the MDA-MB-231 breast cancer cell line with a higher propensity for lung metastasis [16]. LM2-4175 cell line was provided by Joan Massagué from Memorial Sloan-Kettering Cancer Center. MDA-MB-231 was purchased from Cell Bank/Stem Cell Bank affiliated with the Shanghai Institute of Biochemistry and Cell Biology. MDA-MB-231 and LM2-4175 cells were both cultured in Leibovitz’s L15 medium (Invitrogen) with 10% fetal bovine serum (FBS). MDA-MB-231 cells were serum-starved for 24 h and then treated with TGFβ 5 ng/ml. Recombinant human TGFβ was purchased from Invitrogen (PHG9202).

### Microarray analysis

The changed genes in MDA-MB-231 cells after TGFβ treatment and the differentially expressed genes in MDA-MB-231 cells compared with LM2-4175 cells were determined using Human Genome-U133 Plus 2.0 array (Affymetrix, Santa Clara, CA). The microarray analysis was carried out according to the standard protocol [17]. Briefly, total RNA was purified, amplified, and labeled with biotin. The fragmented, biotinylated cDNA was hybridized with the microarray and scanned. Raw gene expression levels were normalized using the Robust Multi-array Averaging (RMA) method in ‘affy’ package R software. Raw expression data were annotated with GPL570. The Volcano plots were generated using ‘ggplot2’ package. The CEL and matrix data of MDA-MB-231 and LM2-4175 cells were available at gene expression omnibus (GEO) datasets with accession number GSE184828.

### Data sources

The Cancer Genome Atlas (TCGA) datasets were collected from the UCSC Xena hub (https://tcga.xenahubs.net) [18]. The expressions of breast cancer cell lines were downloaded from the Cancer Cell Line Encyclopedia [19, 20]. E-MTAB-365 dataset was downloaded from https://www.ebi.ac.uk/arrayexpress/website [21]. Other datasets were collected from the GEO database (www.ncbi.nlm.nih.gov/geo). The detailed references of published datasets used in this study were provided in Supplementary data. All the datasets were processed using R software. All the samples used in this study were derived from primary untreated breast tumors.

### Univariable Cox regression and lung metastasis-free survival analysis

Raw CEL files of 404 breast cancer patients from GSE2034 [22], GSE2603 [16], and GSE5327 [23] three independent datasets were normalized using the RMA method. The normalized expressions of breast cancer patients were used for further lung metastasis-free survival analysis through univariable Cox regression. Univariable Cox regression analysis was carried out using “coxph” method in “survival” and “survminer” packages. Forest plots were generated using “ggforest” and “forestplot” packages. Hazard ratio (HR) and *P* values were determined using Cox regression survival analysis. Lung metastasis-free survival analysis was carried out using “survival” and “survminer” packages. *P* values were determined by the log-rank test.

### Kaplan–Meier survival analysis

R software ‘survival’ and “survminer” packages were used for Kaplan–Meier survival analysis. *P* values were determined by the log-rank test. Breast cancer patients were divided into ‘high’ and ‘low’ gene expression sub-groups based on the optimal cutoff points using the “survminer” package.

### Immunohistochemistry

The protein expression levels of TGFBI in 139 Chinese breast cancer tissues were detected by immunohistochemistry using commercial tissue microarray from Shanghai OUTDO Biotech. All the samples used in this study were derived from primary untreated breast tumors. Rabbit anti-TGFBI antibody was purchased from Cell Signaling Technology. Immunohistochemistry was carried out according to the previously described standard protocol [24]. The expression intensity of TGFBI was determined in a blinded manner. The prognosis of expression intensity of TGFB was determined using ‘survival’ and “survminer” packages in R software.

### Tumor microenvironment estimation and single sample gene set enrichment analysis (ssGSEA)

The stromal scores and immune scores of primary breast cancer patients were determined by ‘ESTIMATE’ package in R software based on ssGSEA assay [25]. ssGSEA assay was carried out using “GSVA” package in R software [25]. “GSVA” in the ssGSEA was used to evaluate the enrichment of stromal scores and immune scores in each sample based on the expression of stromal and immune-related genes.

### Statistical analysis

Statistical analysis was performed through paired Student’s *t* test.

## Results

### The prognosis of TGFβ regulated genes in ER-negative breast cancer

**Fig. 1:**
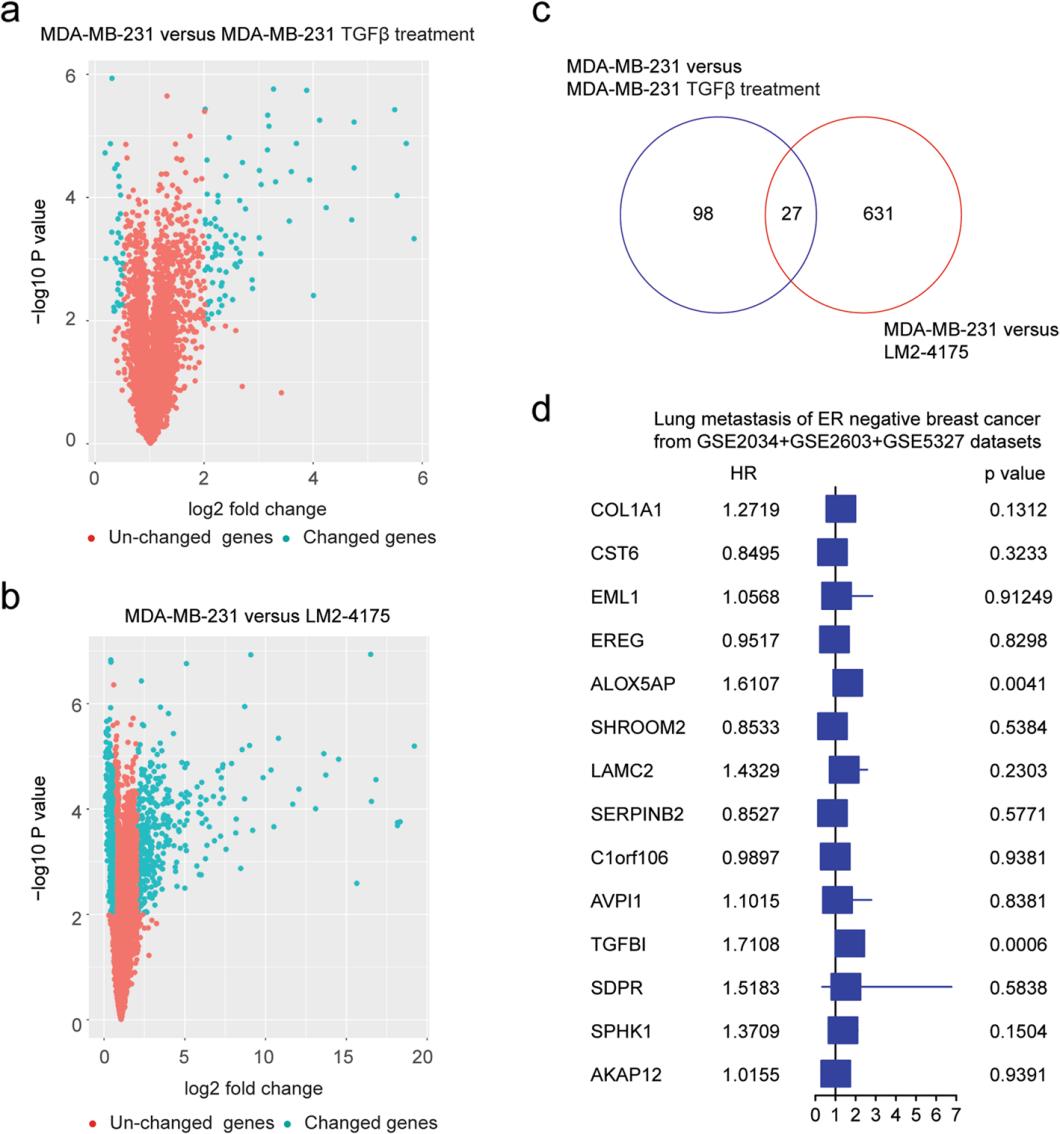
The prognosis of TGFβ regulated genes in ER-negative breast cancer. **a** Volcano plot showed the changed genes in MDA-MB-231 cells treated with TGFβ. **b** Contrast with MDA-MB-231 cells, the differentially expressed genes in LM2-4175 cells. **c** Overlapped differentially expressed genes in MDA-MB-231 cells treated with TGFβ and in LM2-4175 cells. **d** Forest plot revealed the associations of TGFβ regulated genes with lung metastasis-free survival in ER-negative breast cancer

MDA-MB-231 is a triple-negative breast cancer cell line and is used for the studies of breast cancer metastasis and the lung metastasis of MDA-MB-231 cells is correlated with TGFβ signal [16]. First, the TGFβ regulated genes in MDA-MB-231 cells were identified through gene expression microarray. Compared with the untreated control, 125 genes were changed after TGFβ treatment in MDA-MB-231 cells based on the thresholds of *P* value < 0.001 and fold change > 2 (Fig. 1a).

LM2-4175 is a sub-population of MDA-MB-231 cells with increased expressions of lung metastasis signature and a high propensity to form pulmonary nodules [16]. Compared with parental MDA-MB-231 cells, 658 genes were differentially expressed in LM2-4175 cells with *P* value < 0.001 and fold change > 2 thresholds (Fig. 1b). Moreover, 27 genes were both changed in LM2-4175 cells and were regulated by TGFβ in MDA-MB-231 cells (Fig. 1c).

The prognosis of those 27 genes was determined using univariable Cox regression analysis. In total, 404 breast cancer patients with expression and clinical lung metastasis were obtained from the combination of GSE2034, GSE2603, and GSE5327 datasets, including 164 ER-negative patients and 240 ER-positive patients. Because of the different microarray platforms, only COL1A1, CST6, EML1, EREG, ALOX5AP, SHROOM2, LAMC2, SERPINB2, C1orf106, AVPI1, TGFBI, SDRP, SPHK1, and AKAP12 were detected and only two genes ALOX5AP and TGFBI were correlated with the lung metastasis of ER-negative breast cancer (Fig. 1d). The prognosis of ALOX5AP was revealed by multiple studies [26, 27]. However, the prognosis of TGFBI was not yet clear in breast cancer.

### TGFBI is highly expressed in metastatic breast cancer cells and associated with the lung metastasis of breast cancer

**Fig. 2:**
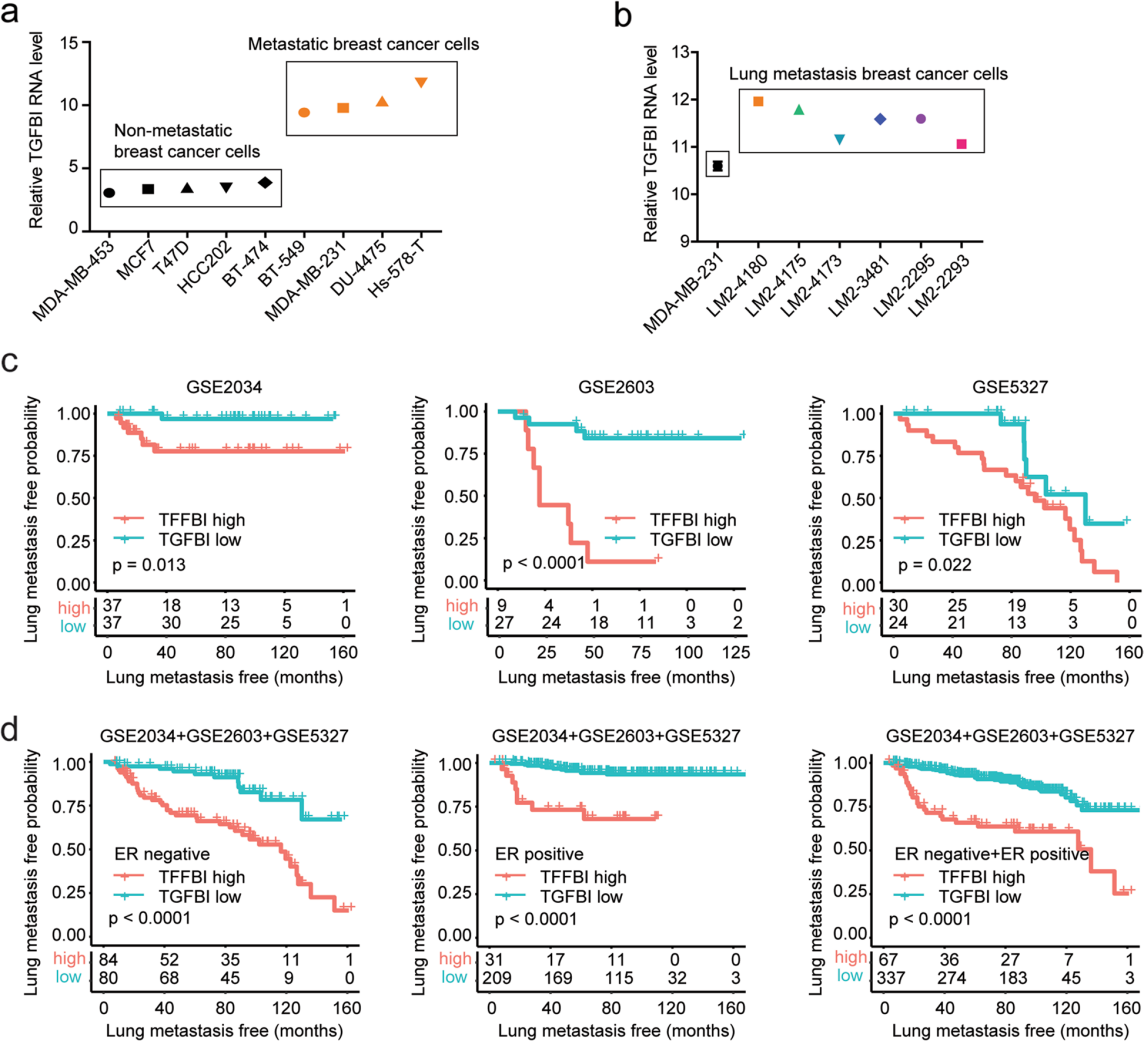
TGFBI is highly expressed in metastatic breast cancer cells and associated with the lung metastasis of breast cancer. **a** Expressions of TGFBI in non-metastatic breast cancer cells and metastatic breast cells. **b** Expressions of TGFBI in MDA-MB-231 cells and MDA-MB-231 derived lung metastasis LM2 cells. **c** Lung metastasis-free survival of ER-negative breast cancer patients with higher TGFBI expression or lower TGFBI expression in GSE2034, GSE2603, and GSE5327 datasets. **d** The prognosis of TGFBI in ER-negative, ER-positive, and all breast cancer patients in the combined GSE2034, GSE2603, and GSE5327 cohort

First, the expressions of TGFBI were analyzed in various breast cancer cell lines. Compared with non-metastatic breast cancer cells MDA-MB-453, MCF7, T47D, HCC202, and BT-474, the expression levels of TGFBI were higher in metastatic breast cancer cells BT-549, MDA-MB-231, DU-4475, and Hs-578-T (Fig. 2a). Also, compared with parental MDA-MB-231 cells, the expression levels of TGFBI were higher in LM2-4180, LM2-4175, LM2-4173, LM2-3481, LM2-2295, and LM2-2293 breast cells with specific higher lung metastasis (Fig. 2b).

Moreover, the correlations of TGFBI with lung metastasis of breast cancer were further determined using Kaplan–Meier survival analysis. In each GSE2034, GSE2603, and GSE5327 dataset, TGFBI higher expression was associated with a higher probability of lung metastasis in ER-negative breast cancer patients (Fig. 2c). Furthermore, combining the expression data of GSE2034, GSE2603 and GSE5327 datasets into a larger cohort, we found that in both ER negative and ER positive breast cancer patients, TGFBI higher expression was associated with the higher probability of lung metastasis (Fig. 2d). Also, in those 404 breast cancer patients, TGFBI higher expression was associated with the higher probability of lung metastasis (Fig. 2d).

### TGFBI is associated with poor clinical outcomes of breast cancer

**Fig. 3:**
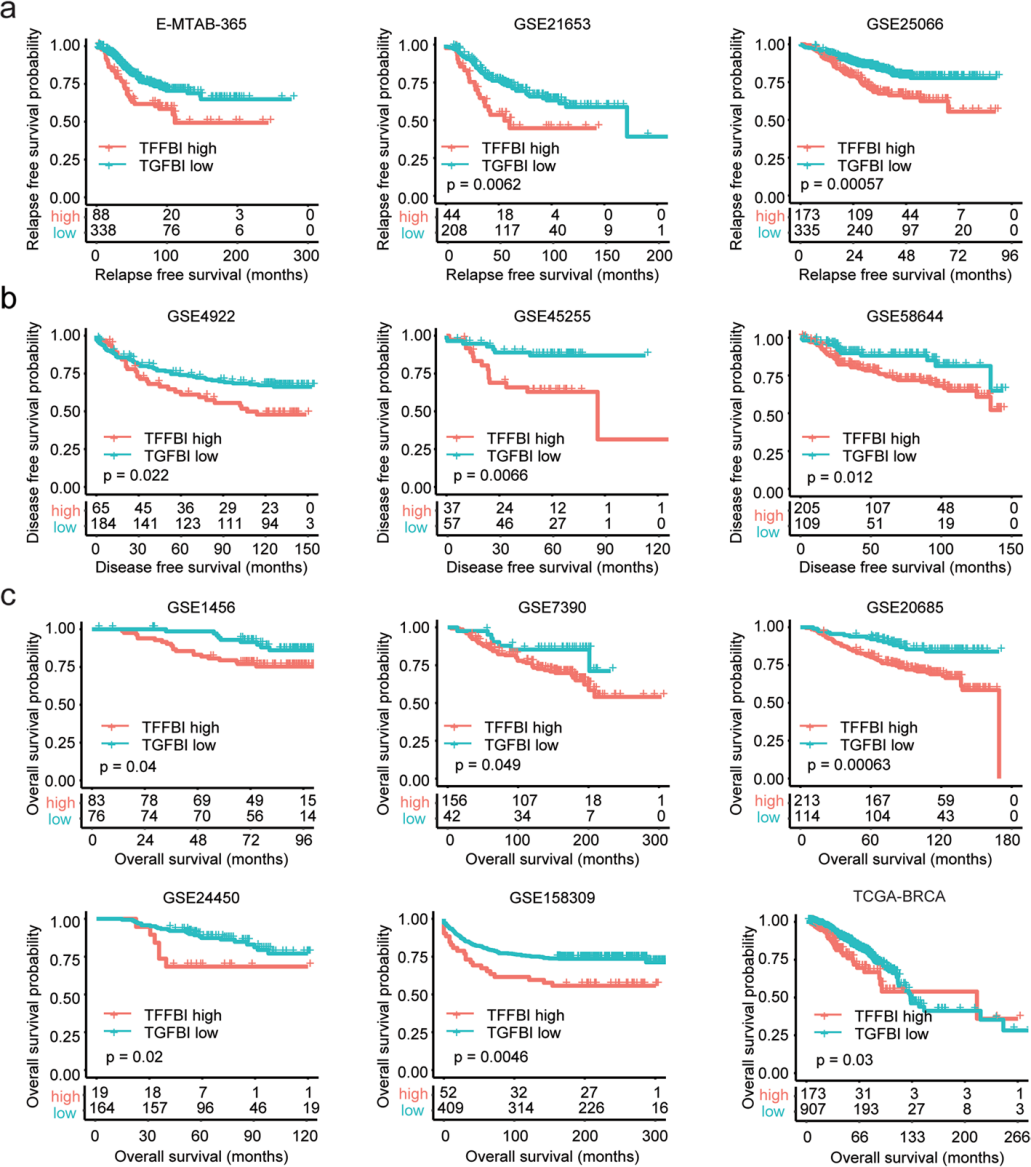
TGFBI is associated with poor clinical outcomes of breast cancer. **a** Correlations of TGFBI expression with breast cancer relapse-free survival in E-MATB-365, GSE21653, and GSE25066 datasets. **b** Correlations of TGFBI expression with breast cancer disease-free survival in GSE4922, GSE45255, and GSE58644 datasets. **c** Correlations of TGFBI expression with breast cancer overall survival in GSE1456, GSE7390, GSE20685, GSE24450, GSE158309, and TCGA-BRCA datasets

The prognosis of TGFBI in breast cancer was further validated using multiple independent breast cancer cohorts from the European Bioinformatics Institute (EMBL-EBI), GEO, and TCGA datasets. In E-MTAB-365, GSE21653, and GSE25066 datasets, higher expression of TGFBI was associated with the shorted relapse-free survival of breast cancer (Fig. 3a). Moreover, in GSE4922, GSE45255, and GSE58644 datasets, higher expression of TGFBI was associated with the shorted disease-free survival of breast cancer (Fig. 3b). Furthermore, in GSE1456, GSE7390, GSE20685, GSE24450, and GSE158309 datasets, but not in TCGA-BRCA dataset, higher expression of TGFBI was associated with the shorted overall survival of breast cancer (Fig. 3c). Those results suggested the poor prognosis of TGFBI in breast cancer.

### The prognosis of TGFBI in Chinese breast cancer patients

**Fig. 4:**
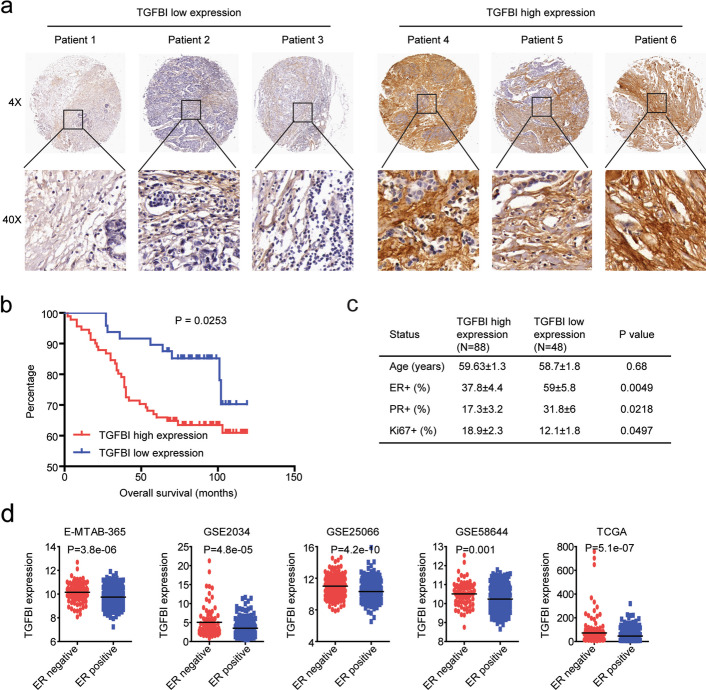
The prognosis of TGFBI in Chinese breast cancer patients. **a** Representative images of immunohistochemistry showed the high expressions of TGFBI and low expressions of TGFBI in Chinese breast cancer tissues. **b** Overall survival of breast cancer patients with higher TGFBI expressions or lower TGFBI expressions. **c** ER, PR, and Ki67 statuses of Chinese breast cancer patients with high expressions of TGFBI and low expressions of TGFBI. **d** Expressions of TGFBI in ER-negative or ER-positive breast cancer patients in E-MTAB-365, GSE2034, GSE25066, GSE58644, and TCGA-BRCA datasets

Next, we validated the prognosis of TGFBI using breast cancer tissue microarray. In total, the expressions of TGFBI in 139 Chinese breast cancer tissues were analyzed by immunohistochemistry. TGFBI expression could be detected in most breast cancer tissues. Based on TGFBI expression levels, breast cancer patients were classified into the TGFBI high expression sub-group and the TGFBI low expression sub-group (Fig. 4a). We found that high expression of TGFBI was correlated with the poor prognosis of breast cancer patients. Breast cancer patients with low expression of TGFBI had significantly longer overall survival (Fig. 4b).

Furthermore, we analyzed the expressions of TGFBI in breast cancer patients with different ER and PR statuses and found that TGFBI was associated with ER and PR expressions in breast cancer. In 88 TGFBI highly expressed breast cancer patients, the percentage of ER + was 37.8 ± 4.4, while, in 48 TGFBI lowly expressed breast cancer patients, the percentage of ER + was 59.58 ± 5.8 (Fig. 4c). In TGFBI highly expressed breast cancer patients, the percentage of PR + was 17.3 ± 3.2, while, in TGFBI lowly expressed breast cancer patients, the percentage of PR + was 31.8 ± 6 (Fig. 4c). Also, TGFBI highly expressed breast cancer patients were with higher ki67 expressions (Fig. 4c).

The associations of TGFBI with ER status in breast cancer patients were further validated using EMBL-EBI, GEO, and TCGA-BRCA datasets. In E-MTAB-365, GSE2034, GSE25066, GSE58644, and TCGA-BRCA datasets, the expressions of TGFBI were significantly higher in ER-negative breast cancer patients than in ER-positive breast cancer patients (Fig. 4d).

### TGFBI methylation level is associated with the clinical outcomes of breast cancer

**Fig. 5:**
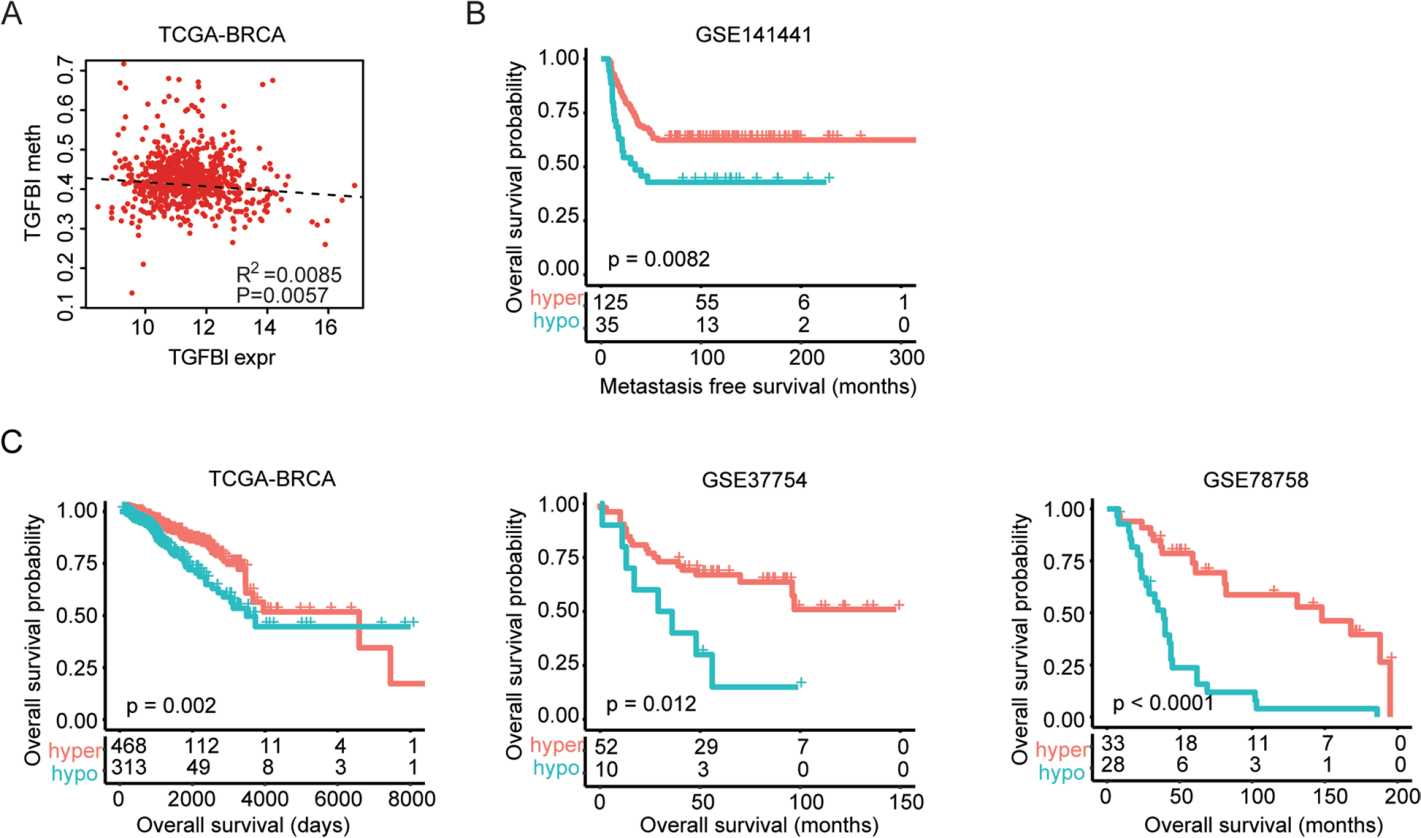
TGFBI methylation level is associated with the clinical outcomes of breast cancer. **a** The correlations of TGFBI methylation and TGFBI expression in the TCGA-BRCA dataset. **b** Correlations of TGFBI methylation with breast cancer metastasis-free survival in GSE141441 dataset. **c** Correlations of TGFBI methylation with breast cancer overall survival in GSE37745, GSE78758, and TCGA-BRCA datasets

The high expressions of TGFBI in some cases of breast cancer may be induced by TGFβ. However, the alterations of TGFBI methylation may also contribute to the changed expressions of TGFBI. In the TCGA-BRCA dataset, TGFBI expression levels were significantly correlated with TGFBI methylation levels (Fig. 5a). Next, we determined the prognostic relevance of TGFBI methylation levels in breast cancer. Corresponding to the poor prognosis of TGFBI expression, TGFBI methylation was associated with the good prognosis of breast cancer. In the GSE141441 dataset, hypo-methylation of TGFBI was correlated with the shorted metastasis-free survival (Fig. 5b). Furthermore, in TCGA-BRCA, GSE37754, and GSE78758 datasets, hypo-methylation of TGFBI was correlated with the shorted overall survival of breast cancer (Fig. 5c).

### Identification of the signaling pathways associated with TGFBI expression

**Fig. 6:**
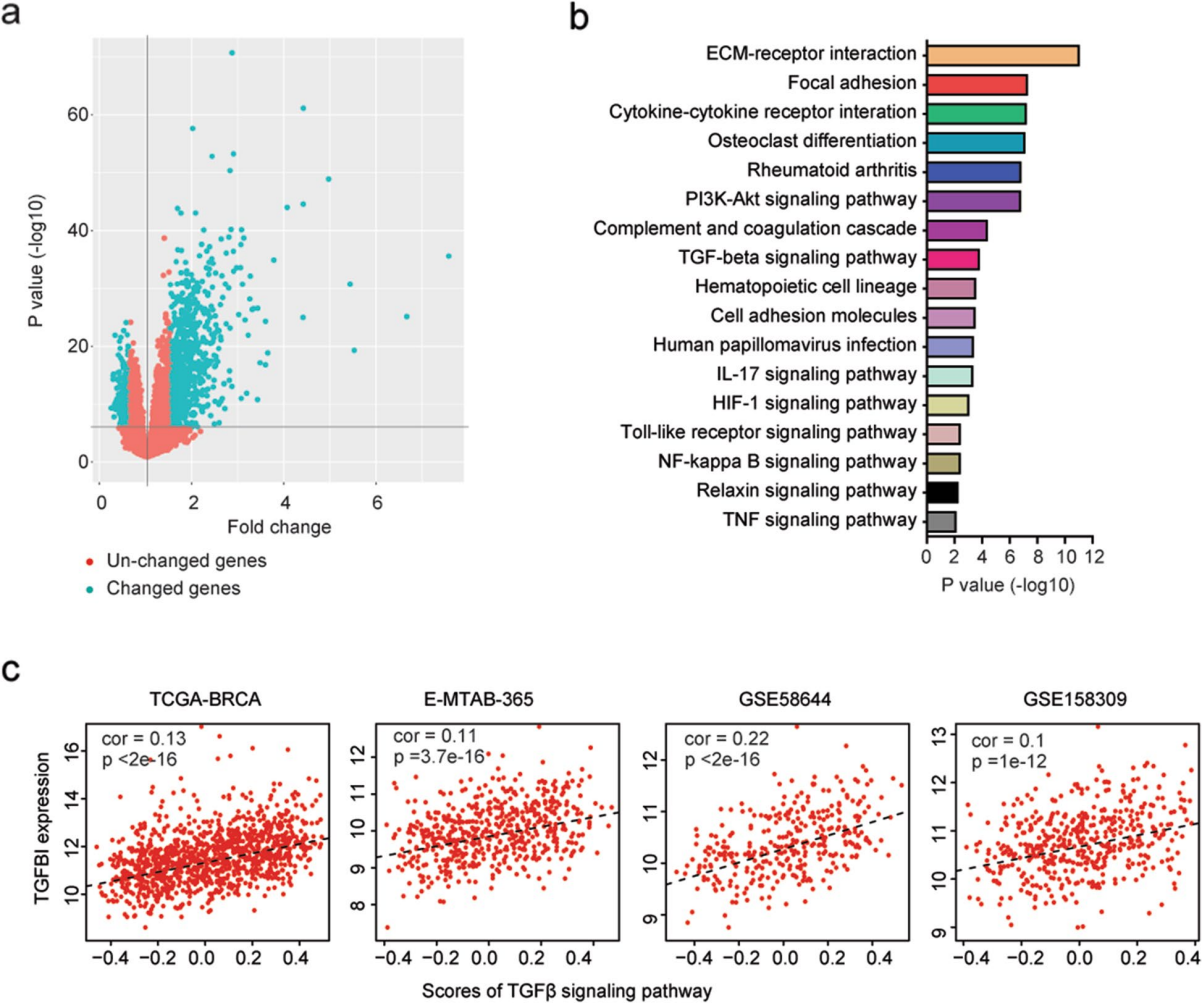
Identification of the signaling pathways associated with TGFBI expression. **a** Volcano plot showed the changed genes in TGFBI highly expressed breast cancer patients in the TCGA-BRCA dataset. **b** Signaling pathways associated with TGFBI in breast cancer patients were determined. **c** The correlations of TGFBI expression and TGFβ signaling pathway in TCGA-BRCA, E-MTAB-365, GSE58644, and GSE158309 datasets

To further understand the prognostic effects of TGFBI in breast cancer, genes differentially expressed in breast cancer patients with higher TGFBI expressions were identified in the TCGA-BRCA dataset. Compared with breast cancer patients with lower TGFBI expressions, 1253 genes were differentially expressed in breast cancer patients with higher TGFBI expressions in TCGA-BRCA dataset based on the thresholds of *P* value < 0.001 and fold change > 1.5 (Fig. 6a). Those genes were significantly correlated with ECM-receptor interaction, focal adhesion, PI3K-Akt signaling pathway, HIF1 signaling pathway and TGFβ signaling pathway (Fig. 6b).

The correlations of TGFBI with the TGFβ signaling pathway were further analyzed. The scores of the TGFβ signaling pathway were determined using ssGSEA assay. We found that TGFBI was significantly correlated with the TGFβ signaling pathway in TCGA-BRCA, E-MTAB-365, GSE58644, and GSE158309 datasets (Fig. 6c).

### Correlations of TGFBI and immune infiltrations of breast cancer

**Fig. 7:**
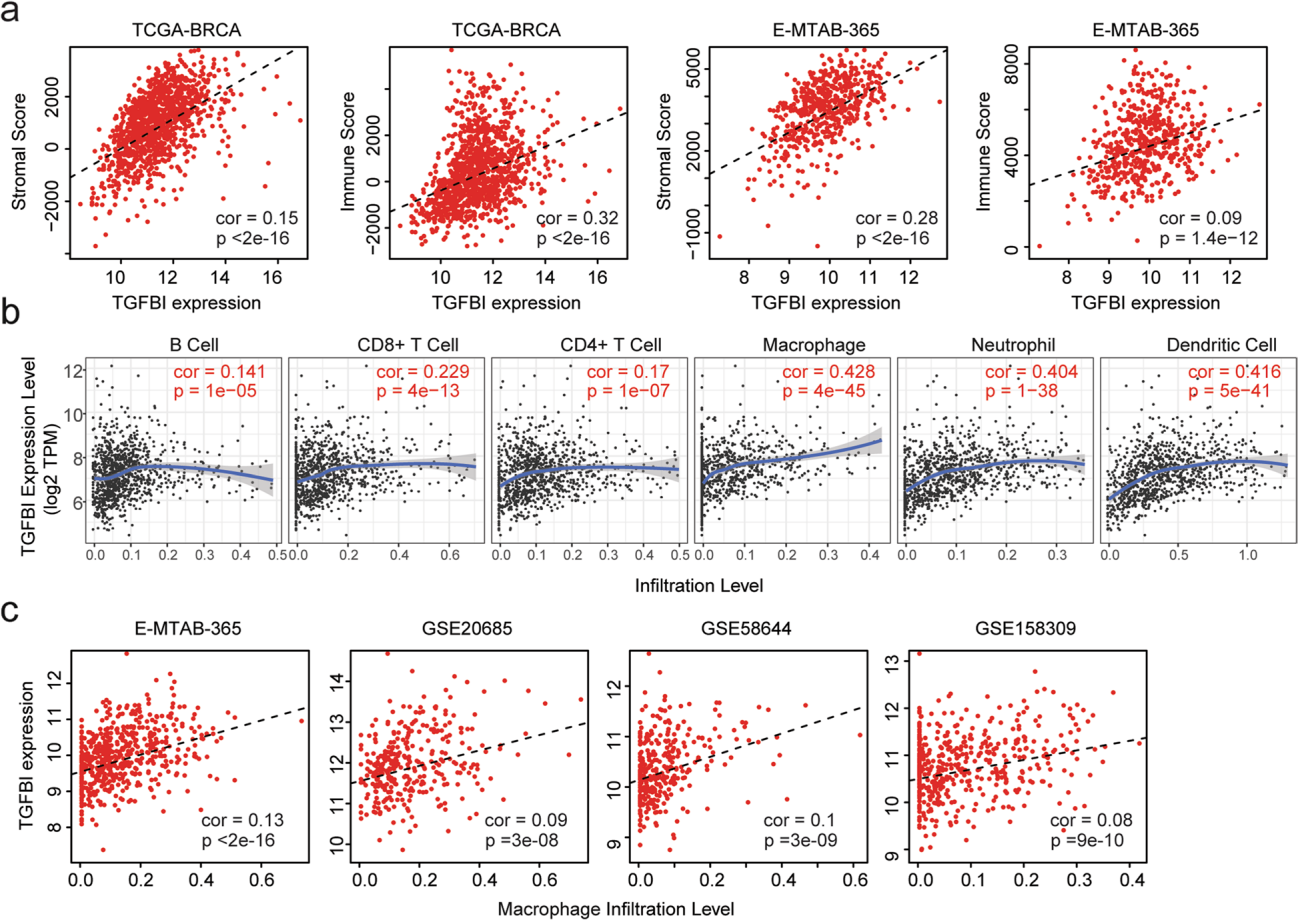
Correlations of TGFBI and immune infiltrations of breast cancer. **a** Correlations of TGFBI with stromal and immune scores in TCGA-BRCA and E-MTAB-365 datasets. **b** Correlations of TGFBI and the infiltrations of six immune-related cell types in breast cancer patients in TCGA-BRCA dataset. **c** Correlations of TGFBI and the infiltrations of macrophages in E-MTAB-365, GSE20685, GSE58644, and GSE158309 datasets

TGFβ affects the infiltrations of immune cells in the tumor microenvironment. So, we tested the correlations between TGFBI and immune infiltrations of breast cancer. The stromal score and immune score of breast cancer were calculated through “ESTIMATE” algorithm in TCGA-BRCA and E-MTAB-365 datasets. The stromal scores as well as the immune scores were both associated with TGFBI expression levels in TCGA-BRCA and E-MTAB-365 datasets (Fig. 7a).

Moreover, in “TIMER” database, the macrophages infiltrations were most correlated with TGFBI expressions in breast cancer patients (Fig. 7b). Infiltrations of other immune-related cells, like neutrophil cells and dendritic cells were also correlated with TGFBI expressions in “TIMER” database (Fig. 7b). Furthermore, in E-MTAB-365, GSE20685, GSE58644 and GSE158309 datasets, the infiltrations of macrophages were all statistically correlated with TGFBI expressions in breast cancer patients (Fig. 7c).

## Discussion

The lung metastasis of ER-negative breast cancer cells is required of TGFβ [7]. However, for the 125 TGFβ regulated genes in MDA-MB-231 cells, only 27 genes were differentially expressed in LM2-4175 cells. Moreover, only two genes ALOX5AP and TGFBI were correlated with the lung metastasis of ER-negative breast cancer. Those results highlighted the multifunctional property of TGFβ in the regulations of breast cancer metastasis. Although TGFβ is specifically related to lung metastasis in ER-negative breast cancer [7], TGFβ also regulates the bone metastasis [28, 29] and brain metastasis [30] of breast cancer.

TGFBI is a conservative cell matrix protein and plays important roles in histomorphogenesis and mesodermal differentiation [31]. Like TGFβ, TGFBI either inhibits or promotes tumorigenesis depending on the different types of cancers [32]. TGFBI is lowly expressed in breast cancer, ovarian cancer, and lung cancer [33, 34], but highly expressed in clear cell renal cancer and colorectal cancer [35, 36]. TGFBI could inhibit the growth of breast cancer cells [37] and suppress breast cancer metastasis by inhibiting matrix metalloproteinase activity [38]. On the contrary, we showed that TGFBI was highly expressed in metastatic breast cancer cells and associated with higher lung metastasis of breast cancer. However, in the TCGA-BRCA dataset, higher expression of TGFBI was associated with the shorted overall survival of breast cancer. Those differences highlighted the complexity of breast cancer and results derived from one cohort may not be replicated in other cohorts. So, we used as many breast cancer cohorts and tissue microarrays to get a more precise conclusion that higher expression of TGFBI was associated with short overall survival of breast cancer.

The high expressions of TGFBI in some cases of breast cancer may be induced by TGFβ. However, except for TGFβ induction, TGFBI is also regulated by transcription factors Sp1 and Sp3 [39]. In HER2-positive breast cancer cells, hyper-methylation mediated the low TGFBI expression and the induction of Herceptin resistance [40]. Consistent with those results, we showed that the alterations of TGFBI methylation may also contribute to the changed expressions of TGFBI. Corresponding with a poor prognosis of TGFBI expression, TGFBI methylation was associated with a good prognosis of breast cancer.

TGFβ affects the infiltrations of immune cells into the tumor microenvironment and is associated with PD-1 checkpoint blockade therapy. Inhibition of the TGFβ signaling pathway enhances the benefits of immune therapies [41, 42]. Like TGFβ, TGFBI also affects the infiltrations of immune cells to generate a suitable tumor microenvironment for breast cancer cell metastasis [43]. Moreover, the functions and downstream mechanisms of TGFBI in lung metastasis of breast cancer were revealed in our study. We showed that TGFBI was correlated with ECM-receptor interaction, focal adhesion, PI3K-Akt signaling pathway, HIF1 signaling pathway, and TGFβ signaling pathway. Also, the stromal scores as well as the immune scores of breast cancer were associated with TGFBI expression levels. Infiltrations of other immune-related cells, like macrophages, neutrophil cells, and dendritic cells were also correlated with TGFBI expressions.

Overall, we highlighted that TGFβ induced gene TGFBI was correlated with the prognosis and immune infiltrations of breast cancer. Although, in both bioinformatics analysis and tissue microarray validation, our results confirmed the poor prognosis of TGFBI in breast cancer, and the functions of TGFBI associated with the TGFβ signaling pathway and the breast cancer microenvironment, yet detailed functions of TGFBI in the regulations of lung metastasis of breast cancer should be further studied.

## Conclusions

TGFBI was upregulated by TGFβ treatment and over-expressed in LM2-4175 cells. TGFBI was correlated with the prognosis and immune infiltrations of breast cancer.

### Supplementary Information

**Additional file 1.**

## Data Availability

The datasets generated during the current study are available in GEO datasets with accession number GSE184828. The datasets re-analyzed during the current study are available in EMBL-EBI, GEO, and TCGA repository. The references of those datasets are provided in the Supplementary data.

## Abbreviations

TGFβ: Transforming growth factor β
TGFBI: TGFβ-induced gene
ER: Estrogen receptor
PR: Progesterone receptor
HER2: Human epidermal growth factor receptor 2
EMT: Epithelial-mesenchymal transformation
ANGPTL4: Angiopoietin-like 4
MMP2: Metallopeptidase 2
EREG: Epiregulin
VEGFA: Vascular endothelial growth factor A
FBS: Fetal bovine serum
RMA: Robust Multi-array Averaging
GEO: Gene expression omnibus
TCGA: The Cancer Genome Atlas
EMBL-EBI: European Bioinformatics Institute
HR: Hazard ratio
GSEA: Gene set enrichment analysis
