# Supplementary material for: Transforming growth factor-induced gene TGFBI is correlated with the prognosis and immune infiltrations of breast cancer
Source: World J Surg Oncol. 2024 Jan 20;22:22. doi: 10.1186/s12957-024-03301-z (PMC10799375; doi:10.1186/s12957-024-03301-z)
Supplement: Supplementary file 1 — Additional file 1. [file 12957_2024_3301_MOESM1_ESM.docx]

The detailed references of published datasets using in this study:

The TCGA-BRCA datasets [1] and Cancer Cell Line Encyclopedia [2, 3] datasets were used in the study. Datasets used for the analysis of lung metastasis free survival of breast cancer: GSE2034 [4], GSE2603 [5] and GSE5327 [6]. Datasets used for the analysis of relapse free survival of breast cancer: E-MTAB-365 [7], GSE21653 [8, 9] and GSE25066 [10]. Datasets used for the analysis of disease free survival of breast cancer: GSE4922 [11], GSE45255 [12] and GSE58644 [13]. Datasets used for the analysis of overall survival of breast cancer GSE1456, GSE7390 [14, 15], GSE20685 [16], GSE24450 [17-19], GSE158309 [20] and TCGA-BRCA [1]. Datasets used for the analysis of the expression of TGFBI in ER negative or positive breast cancer patients: E-MTAB-365 [7], GSE2034 [4], GSE25066 [10], GSE58644 [13] and TCGA-BRCA [1]. Datasets used for the analysis of the prognosis of TGFBI methylation: GSE141441, GSE37754 [21], GSE78758 [22] and TCGA-BRCA [1]. Datasets used for the analysis of correlations of TGFBI expression and the score of TGFβ signaling pathway: E-MTAB-365 [7], GSE58644 [13], GSE158309 [20] and TCGA-BRCA [1]. Datasets used for the analysis of correlations of TGFBI expression and the infiltrations of macrophage cell: E-MTAB-365 [7], GSE20685 [16], GSE58644 [13] and GSE158309 [20].

**Reference**

1. Cancer Genome Atlas N: **Comprehensive molecular portraits of human breast tumours**. *Nature* 2012, **490**(7418):61-70.

2. Barretina J, Caponigro G, Stransky N, Venkatesan K, Margolin AA, Kim S, Wilson CJ, Lehar J, Kryukov GV, Sonkin D *et al*: **The Cancer Cell Line Encyclopedia enables predictive modelling of anticancer drug sensitivity**. *Nature* 2012, **483**(7391):603-607.

3. Ghandi M, Huang FW, Jane-Valbuena J, Kryukov GV, Lo CC, McDonald ER, 3rd, Barretina J, Gelfand ET, Bielski CM, Li H *et al*: **Next-generation characterization of the Cancer Cell Line Encyclopedia**. *Nature* 2019, **569**(7757):503-508.

4. Wang Y, Klijn JG, Zhang Y, Sieuwerts AM, Look MP, Yang F, Talantov D, Timmermans M, Meijer-van Gelder ME, Yu J *et al*: **Gene-expression profiles to predict distant metastasis of lymph-node-negative primary breast cancer**. *Lancet* 2005, **365**(9460):671-679.

5. Minn AJ, Gupta GP, Siegel PM, Bos PD, Shu W, Giri DD, Viale A, Olshen AB, Gerald WL, Massague J: **Genes that mediate breast cancer metastasis to lung**. *Nature* 2005, **436**(7050):518-524.

6. Minn AJ, Gupta GP, Padua D, Bos P, Nguyen DX, Nuyten D, Kreike B, Zhang Y, Wang Y, Ishwaran H *et al*: **Lung metastasis genes couple breast tumor size and metastatic spread**. *Proc Natl Acad Sci U S A* 2007, **104**(16):6740-6745.

7. Guedj M, Marisa L, de Reynies A, Orsetti B, Schiappa R, Bibeau F, MacGrogan G, Lerebours F, Finetti P, Longy M *et al*: **A refined molecular taxonomy of breast cancer**. *Oncogene* 2012, **31**(9):1196-1206.

8. Sabatier R, Finetti P, Cervera N, Lambaudie E, Esterni B, Mamessier E, Tallet A, Chabannon C, Extra JM, Jacquemier J *et al*: **A gene expression signature identifies two prognostic subgroups of basal breast cancer**. *Breast Cancer Res Treat* 2011, **126**(2):407-420.

9. Sabatier R, Finetti P, Adelaide J, Guille A, Borg JP, Chaffanet M, Lane L, Birnbaum D, Bertucci F: **Down-regulation of ECRG4, a candidate tumor suppressor gene, in human breast cancer**. *PLoS One* 2011, **6**(11):e27656.

10. Itoh M, Iwamoto T, Matsuoka J, Nogami T, Motoki T, Shien T, Taira N, Niikura N, Hayashi N, Ohtani S *et al*: **Estrogen receptor (ER) mRNA expression and molecular subtype distribution in ER-negative/progesterone receptor-positive breast cancers**. *Breast Cancer Res Treat* 2014, **143**(2):403-409.

11. Ivshina AV, George J, Senko O, Mow B, Putti TC, Smeds J, Lindahl T, Pawitan Y, Hall P, Nordgren H *et al*: **Genetic reclassification of histologic grade delineates new clinical subtypes of breast cancer**. *Cancer Res* 2006, **66**(21):10292-10301.

12. Nagalla S, Chou JW, Willingham MC, Ruiz J, Vaughn JP, Dubey P, Lash TL, Hamilton-Dutoit SJ, Bergh J, Sotiriou C *et al*: **Interactions between immunity, proliferation and molecular subtype in breast cancer prognosis**. *Genome Biol* 2013, **14**(4):R34.

13. Tofigh A, Suderman M, Paquet ER, Livingstone J, Bertos N, Saleh SM, Zhao H, Souleimanova M, Cory S, Lesurf R *et al*: **The prognostic ease and difficulty of invasive breast carcinoma**. *Cell Rep* 2014, **9**(1):129-142.

14. Desmedt C, Piette F, Loi S, Wang Y, Lallemand F, Haibe-Kains B, Viale G, Delorenzi M, Zhang Y, d'Assignies MS *et al*: **Strong time dependence of the 76-gene prognostic signature for node-negative breast cancer patients in the TRANSBIG multicenter independent validation series**. *Clin Cancer Res* 2007, **13**(11):3207-3214.

15. Patil P, Bachant-Winner PO, Haibe-Kains B, Leek JT: **Test set bias affects reproducibility of gene signatures**. *Bioinformatics* 2015, **31**(14):2318-2323.

16. Kao KJ, Chang KM, Hsu HC, Huang AT: **Correlation of microarray-based breast cancer molecular subtypes and clinical outcomes: implications for treatment optimization**. *BMC Cancer* 2011, **11**:143.

17. Heikkinen T, Greco D, Pelttari LM, Tommiska J, Vahteristo P, Heikkila P, Blomqvist C, Aittomaki K, Nevanlinna H: **Variants on the promoter region of PTEN affect breast cancer progression and patient survival**. *Breast Cancer Res* 2011, **13**(6):R130.

18. Muranen TA, Greco D, Fagerholm R, Kilpivaara O, Kampjarvi K, Aittomaki K, Blomqvist C, Heikkila P, Borg A, Nevanlinna H: **Breast tumors from CHEK2 1100delC-mutation carriers: genomic landscape and clinical implications**. *Breast Cancer Res* 2011, **13**(5):R90.

19. Peurala H, Greco D, Heikkinen T, Kaur S, Bartkova J, Jamshidi M, Aittomaki K, Heikkila P, Bartek J, Blomqvist C *et al*: **MiR-34a expression has an effect for lower risk of metastasis and associates with expression patterns predicting clinical outcome in breast cancer**. *PLoS One* 2011, **6**(11):e26122.

20. Heimes AS, Hartner F, Almstedt K, Krajnak S, Lebrecht A, Battista MJ, Edlund K, Brenner W, Hasenburg A, Sahin U *et al*: **Prognostic Significance of Interferon-gamma and Its Signaling Pathway in Early Breast Cancer Depends on the Molecular Subtypes**. *Int J Mol Sci* 2020, **21**(19).

21. Terunuma A, Putluri N, Mishra P, Mathe EA, Dorsey TH, Yi M, Wallace TA, Issaq HJ, Zhou M, Killian JK *et al*: **MYC-driven accumulation of 2-hydroxyglutarate is associated with breast cancer prognosis**. *J Clin Invest* 2014, **124**(1):398-412.

22. Mathe A, Wong-Brown M, Locke WJ, Stirzaker C, Braye SG, Forbes JF, Clark SJ, Avery-Kiejda KA, Scott RJ: **DNA methylation profile of triple negative breast cancer-specific genes comparing lymph node positive patients to lymph node negative patients**. *Sci Rep* 2016, **6**:33435.
